# MSC-ACE2 Ameliorates *Streptococcus uberis*-Induced Inflammatory Injury in Mammary Epithelial Cells by Upregulating the IL-10/STAT3/SOCS3 Pathway

**DOI:** 10.3389/fimmu.2022.870780

**Published:** 2022-05-23

**Authors:** Shuping Yan, Chonghao Zhang, Xiaoxia Ji, Gang Wu, Xinhe Huang, Yafeng Zhang, Yuanshu Zhang

**Affiliations:** Key Laboratory of Animal Physiology and Biochemistry, Ministry of Agriculture, College of Veterinary Medicine, Nanjing Agricultural University, Nanjing, China

**Keywords:** *S. uberis*, MSC-ACE2, inflammatory injury, pyroptosis, blood-milk barrier, mammary epithelial cells

## Abstract

In the dairy industry, *Streptococcus uberis* (*S. uberis*) is one of the most important pathogenic bacteria associated with mastitis in milk-producing cows, causing vast economic loss. To date, the only real effective method of treating and preventing streptococcal mastitis is antimicrobial therapy. In many inflammatory diseases, mesenchymal stem cells (MSCs) and angiotensin-converting enzyme 2 (ACE2) play an anti-inflammatory and anti-injurious role. Accordingly, we hypothesized that MSCs overexpressing ACE2 (MSC-ACE2) would ameliorate the inflammatory injury caused by *S. uberis* in mammary epithelial cells more efficiently than MSC alone. By activating the transcription 3/suppressor of cytokine signaling 3 (IL-10/STAT3/SOCS3) signaling pathway, MSC-ACE2 inhibited the NF-κB, MAPKs, apoptosis, and pyroptosis passways. Moreover, MSC-ACE2 overturned the downregulation of Occludin, Zonula occludens 1 (ZO-1), and Claudin-3 expression levels caused by *S. uberis*, suggesting that MSC-ACE2 promotes the repair of the blood-milk barrier. MSC-ACE2 demonstrated greater effectiveness than MSC alone, as expected. Based on these results, MSC-ACE2 effectively inhibits EpH4-Ev cell’s inflammatory responses induced by *S. uberis*, and would be an effective therapeutic tool for treating streptococcal mastitis.

## Introduction

*S. uberis* is an important pathogen that induces mastitis in cattle, which severely affects milk production and also has a negative impact on animal welfare (1–4). *S. uberis* evade the host immune system by adhering and internalizing into mammary cells (5, 6), thus posing a great challenge in the management of streptococcal mastitis. Our previous study found that *S. uberis* infection of EpH4-Ev cells downregulated ACE2 expression and demonstrated that inflammatory injury in mammary epithelial cells was associated with an imbalance of ACE2, angiotensin 1-7 [Ang- (1–7)], and angiotensin II (Ang II). Therefore, the ACE2 gene is expected to be a target for streptococcal mastitis.

Angiotensin I can be converted to Ang II by the action of the angiotensin-converting enzyme (ACE), which exerts a pro-inflammatory effect (7). The ACE2 enzyme converts Ang II into Ang-(1–7) to inhibit inflammation and damage (7, 8). Our previous study showed that ACE2 exerts anti-inflammatory and anti-damaging effects in LPS-induced inflammation (9). In porcine intestinal epithelial cells, we have demonstrated that ACE2 inhibits lipopolysaccharide (LPS)-induced inflammation *via* the nuclear factor-κB (NF-κB) and mitogen-activated protein kinases (MAPKs) pathways (10).

Mesenchymal stem cells are pluripotent stem cells with multiple biological potentials, such as regeneration, immunomodulation, repair of damaged tissues, home to the site of injury, and other properties (11–13). Currently, mesenchymal stem cell (MSC) treatment positively affects diseases. Acute myocardial infarction, lung injury, stroke, liver failure, and hematologic disorders are some of these conditions (14–19). There is no information on whether MSCs also play an immunomodulatory role in streptococcal mammary gland injury. This study assumed that MSC-ACE2 would exert more potent anti-inflammatory effects and lessen injury in *S.uberis*-induced inflammation in EpH4-Ev cells. Due to their similar anti-inflammatory and anti-injury properties, ACE2 and MSC together have a more significant effect.

## Materials and Methods

### Transmission of Lentiviral Vectors Into MSCs

This research used MSCs from rats obtained from authenticated cell cultures of the National Collection (Shanghai, China). Lentiviral vectors (Genechem Co., Ltd., Shanghai, China) transduction of MSCs and screening of MSCs carrying the ACE2 gene (MSC-ACE2) and GFP markers (MSC-GFP) are described in our previous study (20). Throughout this study, MSC-ACE2 and MSC-GFP were used for generation 5 ~ 10.

### Bacteria and Growth Requirements

*S. uberis* 0140J was purchased from the American Type Culture Center (Manassas, VA, USA). *S. uberis* 0140 J was inoculated into Todd-Hewitt broth (THB) medium containing 2% fetal bovine serum (FBS; Manassas, VA, USA) for 4.5 h at 37°C by an orbital stirrer to the mid-log stage (OD_600_ = 0.4 ~ 0.6).

### An Injury Model for EpH4-Ev cells

Nanjing Agricultural University Professor Jinfeng Miao provided mouse mammary epithelial cells (EpH4-Ev). The mastitis model was established by treating EpH4-Ev cells with MOI (multiplicity of infection) = 10 *S. uberis* for 3 h.

### MSCs and EpH4-Ev Co-Cultured *In Vitro*

A schematic diagram of the model with EpH4-Ev cells and MSCs (MSC, MSC-GFP, or MSC-ACE2) co-cultured is shown in **Additional File 1: Figure 1**. In brief, EpH4-Ev cells were inoculated in the lower chamber of the trans-well (0.4 µm, Corning Inc., NY), and MSCs (MSC-ACE2, MSC-GFP, or MSC) were inoculated in the trans-well’s upper chamber. When EpH4-Ev cells and MSCs were co-cultured for 21 h, *S. uberis* with MOI = 10 was added to EpH4-Ev cells, and the culture was continued for 3 h. Aside from measuring the concentrations of interleukin (IL)-6, tumor necrosis factor-α (TNF-α), IL-Iβ, Ang-(1–7), and Ang II the supernatant of each cell culture was also tested for N-acetyl-β-D-glucosaminidase (NAGase) activity.

### Viable Bacterial Count

Cells were washed 5 times with phosphate buffered solution (PBS) containing 100 mg/mL gentamicin, followed by 5 times with PBS without gentamicin. Cells were digested by trypsin and then lysed using sterile triple-distilled water. The lysate was diluted multiplicatively and spread onto THB medium plates and incubated at 37°C for 12 h. Colony forming unit (CFU) were counted by diffusion plate method (21).

### Analyzing the Concentration of IL-6, TNF-α, IL-Iβ, Ang-(1–7), Ang II, and NAGase Activity

In the supernatant after cell culture, IL-6, TNF-α, IL-Iβ, Ang-(1–7), and Ang II concentrations were measured by ELISA (Hengyuan Biotechnology Co., Shanghai, China). The manufacturer’s instructions were followed for the ELISA test. As a unit of measurement, TNF-α, IL-Iβ, Ang-(1–7), and Ang II were demonstrated as ng/L. In this case, IL-6 was demonstrated as pg/mL. Using the instructions included in the kit purchased from the Nanjing Jiancheng Bioengineering Institute (Nanjing, China), the action of NAGase was determined in the supernatant of the cell culture medium. NAGase activity was measured in units of U/L.

### Apoptosis Assays

Apoptosis of EpH4-Ev cells was detected by flow cytometry according to the operating instructions of Annexin V-FITC Apoptosis Detection Kit (Beyotine Biotechnology, Shanghai, China). Briefly, cells were treated as in the co-culture model. At the end of co-culture, EpH4-Ev cells were digested with trypsin, centrifuged at 1000 g for 5 min, washed once with cold PBS, then 200 μL of annexin V-FITC stock solution and 10 μL of propidium iodide staining solution were added and incubated for 20 min at room temperature in the dark.

### Quantitative Real-Time PCR (qPCR)

The relative transcript levels of IL-6, IL-10, IL-18, TNF-a, IL-Iβ, Apoptosis-associated speck-like protein containing CARD (ASC), Suppressor of cytokine signaling 3 (SOCS3), ZO-1, Claudin-3, and Occludin in EpH4-Ev cells were analyzed by qRT-PCR assay. The methods of obtaining cDNA and PCR and extracting and obtaining total RNA are based on a previous study (22, 23). There is a list of primer sequences in **Table 1** (**Additional File 1**). To calculate raw cycle thresholds (Ct), the relative Ct (2^-ΔΔCt^) method was used with iQ5 Software that detects sequences (Bio-Rad, California, USA).

**Table 1:** The primer sequences of the genes.

Target genes	Primer sequences (5’-3’)
TNF-α	F: TCCCAGGTTCTCTTCAAGGGA
	R: GGTGAGGAGCACGTAGTCGG
IL-6	F: CAAGAAAGACAAAGCCAGAGTC
	R: GAAATTGGGGTAGGAAGGAC
IL-10	F: CCAGGGAGATCCTTTGATGA
	R: CATTCCCAGAGGAATTGCAT
IL-Iβ	F: GCCTCGTGCTGTCGGACCCATA
	R: TGCAGGGTGGGTGTGCCGTCTT
ASC	F: GAAGTGGACGGAGTGCTGGATG
	R: CTTGTCTTGGCTGGTGGTCTCTG
IL-18	F: GGCCGACTTCACTGTACAACCG
	R: GGTCACAAGCCAGTCCTCTTACTTC
SOCS3	F: GCTCCAAAAGCGAGTACCAGC
	R: AGTAGAATCCGCTCTCCTGCAG
ZO-1	F: GGGAGGGTCAAATGAAGACA
	R: GGCATTCCTGCTGGTTACAT
Occludin	F: GTGAGCTGTGATGTGTGTTGAGCT
	R: GTGGGGAACGTGGCCGATATAATG
Claudin-3	F: TTTCTTTGTCCATTCGGCTTG
	R: ACCGTACCGTCACCACTACCA
β-actin	F: TCTGGCACCACACCTTCTA
	R: AGGCATACAGGGACAGCAC

### Western Blot Assays

Western blot assays were performed with reference to the description of previous studies (23). After co-culture for 24 h, the culture medium was discarded and washed twice with cold PBS. Cells were lysed by adding RIPA potent lysate to each well and total cellular protein was obtained by centrifugation. After the total protein concentration was determined with the BCA kit (Thermo Scientific, USA), the proteins were separated by 10% SDS-PAGE and electro-transferred onto PVDF membranes. The membranes were blocked for 2 h at room temperature *via* 5% skim milk or 5% BSA solution and then incubated overnight at 4°C with the target protein primary antibodies. The membranes were washed with TBST (Tris-buffered saline solution consisting of 0.1% Tween-20 solution) and incubated with HRP-labeled secondary antibodies for 2 h at room temperature. After the membranes were washed 5 times with TBST, the expression of the target proteins was detected by chemiluminescence. Image J was used to analyze the relative protein expression levels. Source antibodies: STAT3 (Bioworld, Nanjing, China); Caspase‐3, Bax, and Bcl2 (ABclonal, Wuhan, China); ASC (HuaBio, Hangzhou, China); ERK, p-ERK (Thr202/Tyr204), p38, p-p38 (Thr180/Tyr182) (Cell Signaling Technology, USA); SOCS3 (Beyotime, Shanghai, China); JNK, p-JNK (phospho Thr183/Y185), p65, p-p65 (phospho Ser536) (Proteintech Group, Wuhan, China); p-STAT3 (phospho Ser727), IL-10, NLRP3, IL-Iβ, Gasdermin D (GSDMD), cleaved caspase-1, ZO-1, Claudin-3 (Affinity Biosciences, USA).

### Statistical Analysis

Data are presented as the mean ± standard error of the mean (SEM). The Independent-Samples T-test Compared Through the Means of SPASS 11.0 for Windows (StatSoft, Inc., Tulsa, USA) was applied to the *S. uberis* treatment group and the control group, respectively. We compared *S. uberis’* treatment group against other groups and determined the effects using one-way ANOVA. Statistically, significant *P* values < 0.05 were considered.

## Results

### Effect of MSC-ACE2 on the Secretion Level of Inflammatory Mediators in EpH4-Ev Cells

**Figure 1:**
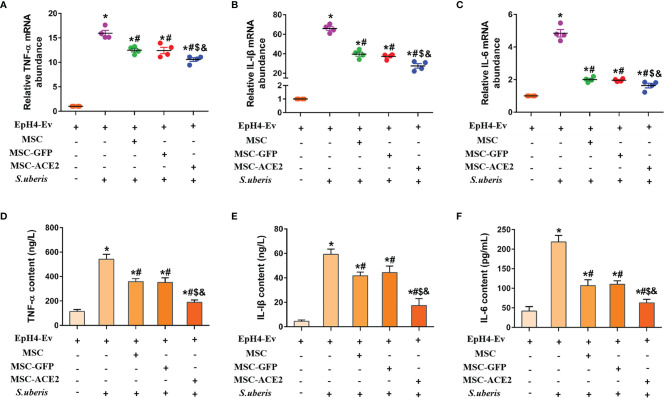
Effect of MSC-ACE2 on the secretion level of inflammatory mediators in EpH4-Ev cells. **(A-C)** Detection of relative transcript levels of TNF-α, IL-1β, and IL-6 in EpH4-Ev cells by qPCR. TNF-α, tumor necrosis factor-α; IL-Iβ, interleukin-Iβ; IL-6, interleukin-6. **(D-F)** Detection of TNF-α, IL-1β, and IL-6 concentration in cell culture supernatants by ELISA. Experiments were repeated three times and data were presented as the mean ± SEM (n = 4). ^*^ *P* < 0.05 vs. EpH4-Ev; ^#^ *P* < 0.05 vs. *S. uberis*; *^$^P* < 0.05 vs. MSC; ^&^ *P* < 0.05 vs. MSC-GFP.

**Figures 1A–C** shows that *S. uberis* infection significantly increased the transcript levels of IL-6, TNF-α, and IL-Iβ in EpH4-Ev cells. However, MSC-GFP and MSC significantly downregulated the transcript levels of IL-6, TNF-α, and IL-Iβ compared with the *S. uberis* infection group. Moreover, MSC-ACE2 further inhibited the upregulation of IL-6, TNF-α, and IL-Iβ transcript levels in EpH4-Ev cells induced by *S. uberis* compared to MSC-GFP and MSC groups. Consistent with expectations, ELISA results (**Figures 1D–F**) showed that *S. uberis* infection significantly upregulated the secretion levels of IL-6, TNF-α, and IL-Iβ in EpH4-Ev cells. However, the secretion levels of IL-6, TNF-α, and IL-Iβ were significantly lower in the MSC-GFP and MSC groups compared with the *S. uberis* infection group. Furthermore, MSC-ACE2 further inhibited the upregulation of IL-6, TNF-α, and IL-Iβ secretion levels in EpH4-Ev cells induced by *S. uberis* compared to MSC-GFP and MSC groups.

### Effect of MSC-ACE2 on NAGase Activity and Bacterial Load in EpH4-Ev Cells

**Figure 2:**
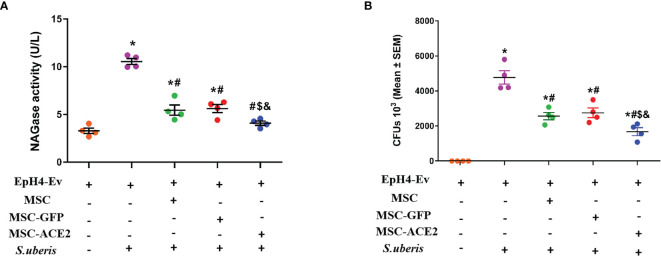
Effect of MSC-ACE2 on NAGase activity and bacterial load in EpH4-Ev cells. **(A)** Detection of NAGase activity in cell culture supernatant according to kit instructions. **(B)**The number of *S. uberis* colonies in EpH4-Ev cells. Experiments were repeated three times and data were presented as the mean ± SEM (n = 4). ^*^ *P* < 0.05 vs. EpH4-Ev; ^#^ *P* < 0.05 vs. *S. uberis*; *^$^P* < 0.05 vs. MSC; ^&^ *P* < 0.05 vs. MSC-GFP.

NAGase activity is used to evaluate mammary epithelial cell injury (24, 25). As shown in **Figure 2A**, *S. uberis* infection resulted in a significant upregulation of NAGase activity in EpH4-Ev cells compared with the control group. When MSC-GFP and MSC were compared with the *S. uberis* infection group, EpH4-Ev cells showed a significant decrease in NAGase activity. MSC-ACE2 further inhibited NAGase activity upregulation caused by *S. uberis* in EpH4-Ev cells when contrasted to MSC-GFP and MSC groups. In addition, we found that MSC, MSC-GFP, and MSC-ACE2 treatments significantly reduced the *S. uberis* load in EpH4-Ev cells and MSC-ACE2 had the best effect (**Figure 2B**).

### The Effects of MSC-ACE2 on EpH4-Ev Cells’ Secretion of Ang II, Ang-(1–7)

**Figure 3:**
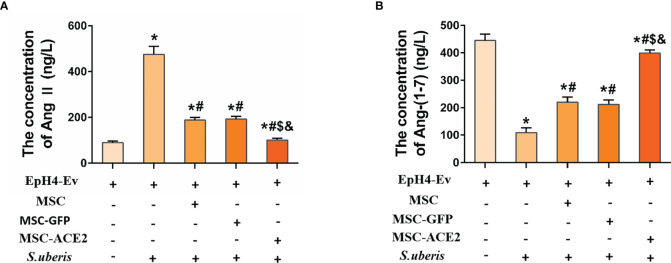
The effects of MSC-ACE2 on EpH4-Ev cells’ secretion of Ang II, Ang-(1–7). **(A)** Detection of Ang II concentration in cell culture supernatants by ELISA. **(B)** Detection of Ang-(1–7) concentration in cell culture supernatants by ELISA. Experiments were repeated three times and data were presented as the mean ± SEM (n = 4). ^*^ *P* < 0.05 vs. EpH4-Ev; ^#^ *P* < 0.05 vs. *S. uberis*; *^$^P* < 0.05 vs. MSC; ^&^ *P* < 0.05 vs. MSC-GFP.

**Figure 3** shows that infection with *S. uberis* caused an increase in Ang II in EpH4-Ev cells and a decrease in Ang-(1–7) levels. MSC-ACE2, MSC-GFP, and MSC groups showed significant downregulation of Ang II and significant upregulation of Ang-(1–7) compared with the *S. uberis* group. Furthermore, the MSC-ACE2 group had a more significant effect than MSC-GFP and MSC groups.

### MSC-ACE2 Inhibited EpH4-Ev Cells Apoptosis Induced by *S. uberis*

**Figure 4:**
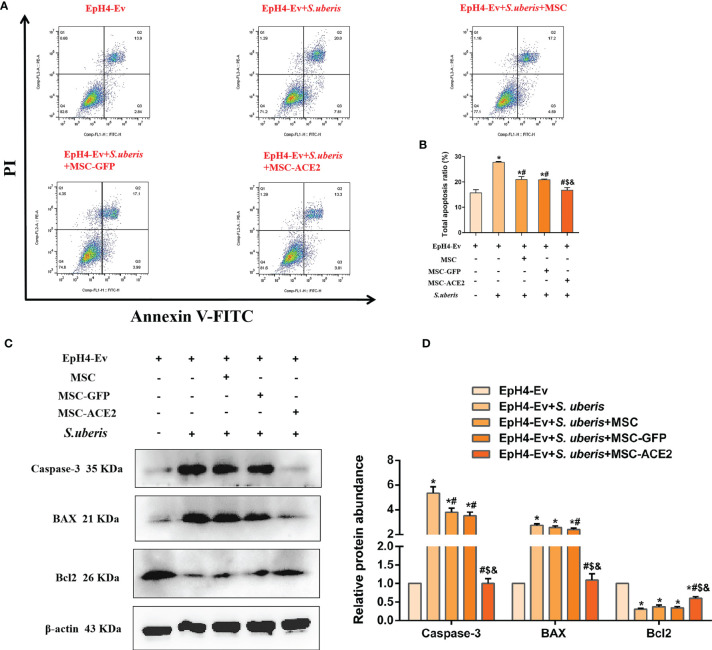
MSC-ACE2 inhibited EpH4-Ev cells apoptosis induced by *S. uberis*. **(A)** Apoptosis kit to detect apoptosis rate. **(B)** Total apoptosis ratio of EpH4-Ev cells. **(C)** Detection of relative protein expression levels of Caspase-3, Bax, and Bcl-2 by Western blot. **(D)** Statistics of the Caspase-3, Bax, and Bcl-2 Western blot results. Experiments were repeated three times and data were presented as the mean ± SEM (n = 3). ^*^ *P* < 0.05 vs. EpH4-Ev; ^#^ *P* < 0.05 vs. *S. uberis*; *^$^P* < 0.05 vs. MSC; ^&^ *P* < 0.05 vs. MSC-GFP.

As shown in **Figure 4**, EpH4-Ev cells infected with *S. uberis* exhibited a significantly higher apoptotic rate than the control group. However, MSC-ACE2, MSC-GFP, and MSC alleviated apoptosis induced by *S. uberis* in EpH4-Ev cells. Moreover, compared with the MSC-GFP and MSC groups, the MSC-ACE2 group had a more significant effect (**Figures 4A, B**). Furthermore, we performed Western blot analyses to determine the relative expression levels of Caspase-3, Bax, and Bcl2 in the apoptosis pathway. Consistent with the expected results, *S. uberis* infection significantly increased the expression levels of Bax and caspase-3, while decreased the expression level of Bcl2. However, MSC-ACE2, MSC-GFP, and MSC inhibited the *S. uberis*-induced upregulation of Caspase-3, Bax, and downregulation of Bcl2. Furthermore, the MSC-ACE2 group had a more significant effect than MSC-GFP and MSC groups (**Figures 4C, D**).

### MSC-ACE2 Ameliorated *S. uberis*-Induced Eph4-Ev Cells Pyroptosis

**Figure 5:**
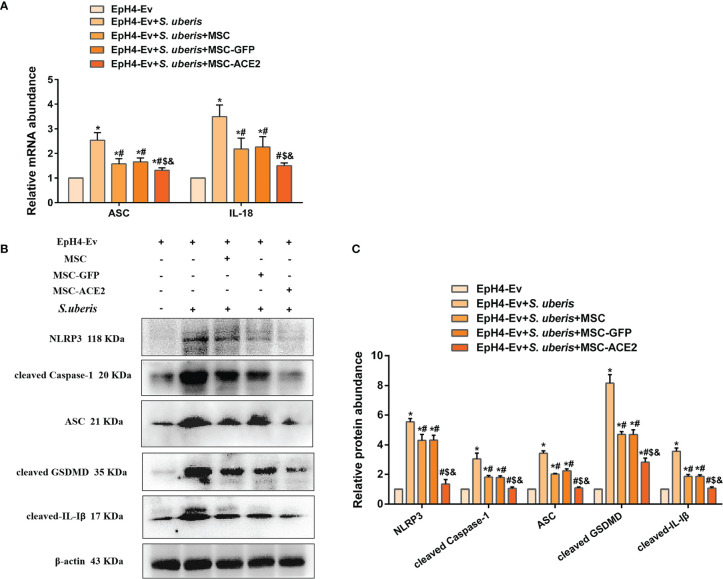
MSC-ACE2 ameliorated *S. uberis*-induced Eph4-Ev cells pyroptosis. **(A)** Detection of relative transcript levels of ASC and IL-18 by qPCR (n = 4). **(B)** Detection of relative protein expression levels of NLRP3, cleaved-Caspase-1 (p20), ASC, cleaved GSDMD, and cleaved-IL-Iβ by Western blot. **(C)** Statistics of NLRP3, cleaved-Caspase-1 (p20), ASC, cleaved GSDMD, and cleaved-IL-Iβ Western blot results (n = 3). Experiments were repeated three times and data were presented as the mean ± SEM. ^*^ *P* < 0.05 vs. EpH4-Ev; ^#^ *P* < 0.05 vs. *S. uberis*; *^$^P* < 0.05 vs. MSC; ^&^ *P* < 0.05 vs. MSC-GFP.

As shown in **Figure 5**, *S. uberis* infection upregulated the expression levels of NLRP3, ASC, cleaved Caspase-1, cleaved GSDMD, and cleaved IL-Iβ. However, MSC-ACE2, MSC-GFP, and MSC substantially inhibited the *S. uberis*-induced upregulation of NLRP3, ASC, cleaved Caspase-1, cleaved GSDMD, and cleaved IL-Iβ expression levels. Moreover, compared to MSC-GFP and MSC, the MSC-ACE2 group had a more significant effect. Consistent with the prediction, qPCR assay results showed that *S. uberis* infection significantly upregulated the transcript levels of ASC, IL-18. Consistent with the expected results, transcript levels of ASC and IL-18 were significantly downregulated in the MSC-ACE2, MSC-GFP, and MSC groups compared to the *S. uberis* infection group. Furthermore, the transcript levels of ASC and IL-18 were further downregulated in the MSC-ACE2 group compared to the MSC-GFP and MSC groups.

### Effect of MSC-ACE2 on IL-10/STAT3 Signaling Pathway

**Figure 6:**
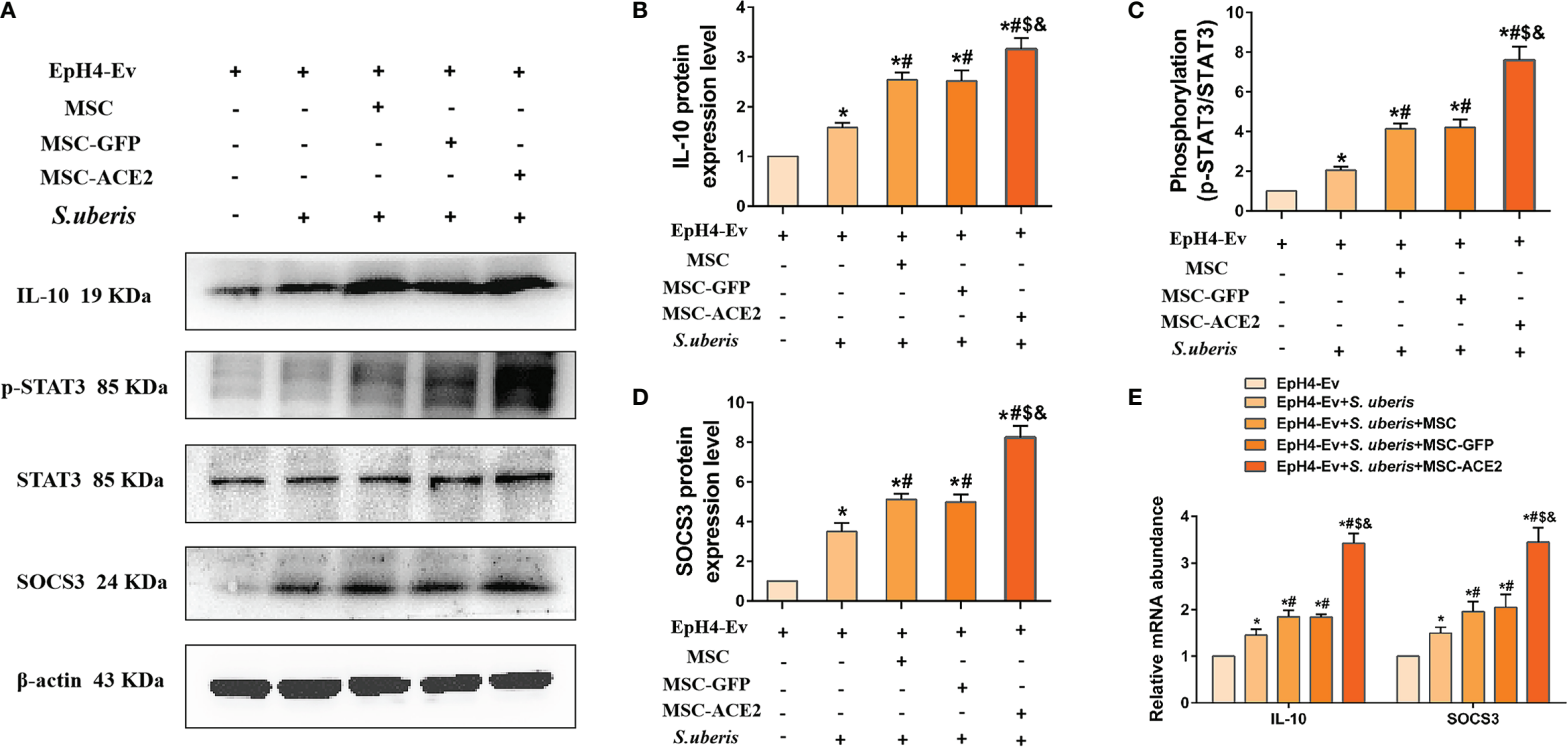
Effect of MSC-ACE2 on IL-10/STAT3/SOCS3 signaling pathway. **(A)** Detection of relative protein expression levels of IL-10, phosphorylation levels of STAT3, STAT3, and SOCS3 by Western blot. **(B-D)** Statistics of the IL-10, phosphorylation levels of STAT3, STAT3, and SOCS3 Western blot results (n = 3). **(E)** Detection of relative transcript levels of IL-10 and SOCS3 in EpH4-Ev cells by qPCR (n = 4). Experiments were repeated three times and data were presented as the mean ± SEM. ^*^ *P* < 0.05 vs. EpH4-Ev; ^#^ *P* < 0.05 vs. *S. uberis*; *^$^P* < 0.05 vs. MSC; ^&^ *P* < 0.05 vs. MSC-GFP.

As shown in **Figure 6**, the relative expression levels of IL-10, p-STAT3, and SOCS3 were significantly upregulated in the *S. uberis* infection group compared with the control group. IL-10, p-STAT3, and SOCS3 were further enhanced by MSC-ACE2, MSC-GFP, and MSC. Furthermore, the MSC-ACE2 group had a more significant effect than MSC-GFP and MSC groups. In addition, the mRNA results of IL-10 and SOCS3 were consistent with the Western blot results.

### Effect of MSC-ACE2 on NF-κB and MAPKs Pathways

**Figure 7:**
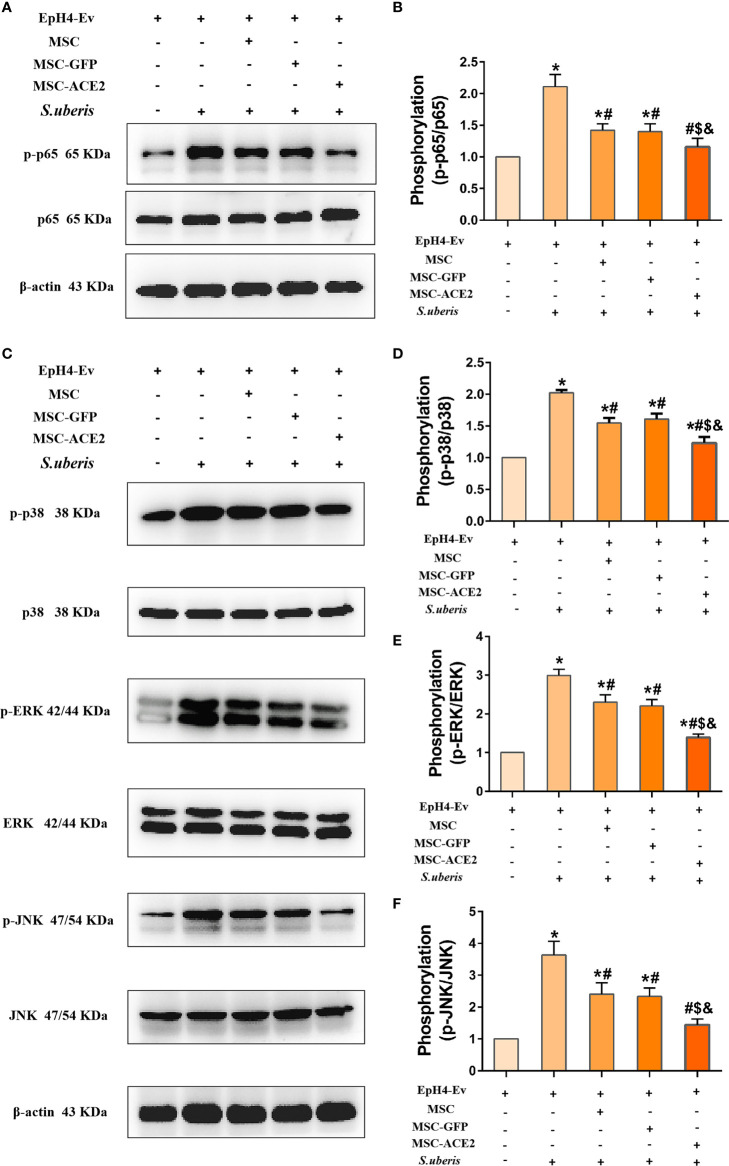
Effect of MSC-ACE2 on NF-κB and MAPKs signaling pathways. **(A)** Detection of relative protein expression levels of p65 and p-p65 by Western blot. **(B)** Statistics of p65 and p-p65 Western blot results. **(C)** Detection of relative protein expression levels of p38, ERK, JNK, and p-p38, p-ERK, p-JNK by Western blot. **(D-F)** Statistics of p38, ERK, JNK, and p-p38, p-ERK, p-JNK Western blot results. Experiments were repeated three times and data were presented as the mean ± SEM (n = 3). ^*^ *P* < 0.05 vs. EpH4-Ev; ^#^ *P* < 0.05 vs. *S. uberis*; *^$^P* < 0.05 vs. MSC; ^&^ *P* < 0.05 vs. MSC-GFP.

The expression abundance of p65, p-p65, ERK, p-ERK, p38, p-p38, JNK, and p-JNK were analyzed by Western blot assay (**Figure 7**). The results showed that *S. uberis* infection significantly upregulated the expression levels of p-p65, p-ERK, p-p38, and p-JNK. Nevertheless, MSC-ACE2, MSC-GFP, and MSC inhibited p-p65, p-ERK, p-p38, and p-JNK expression levels induced by *S. uberis*. Furthermore, the MSC-ACE2 group had a more significant effect compared with the MSC-GFP and MSC groups.

### MSC-ACE2 Reversed the *S. uberis*-Induced Downregulation of the Expression Abundance of blood-Milk Barrier-Associated Proteins

**Figure 8:**
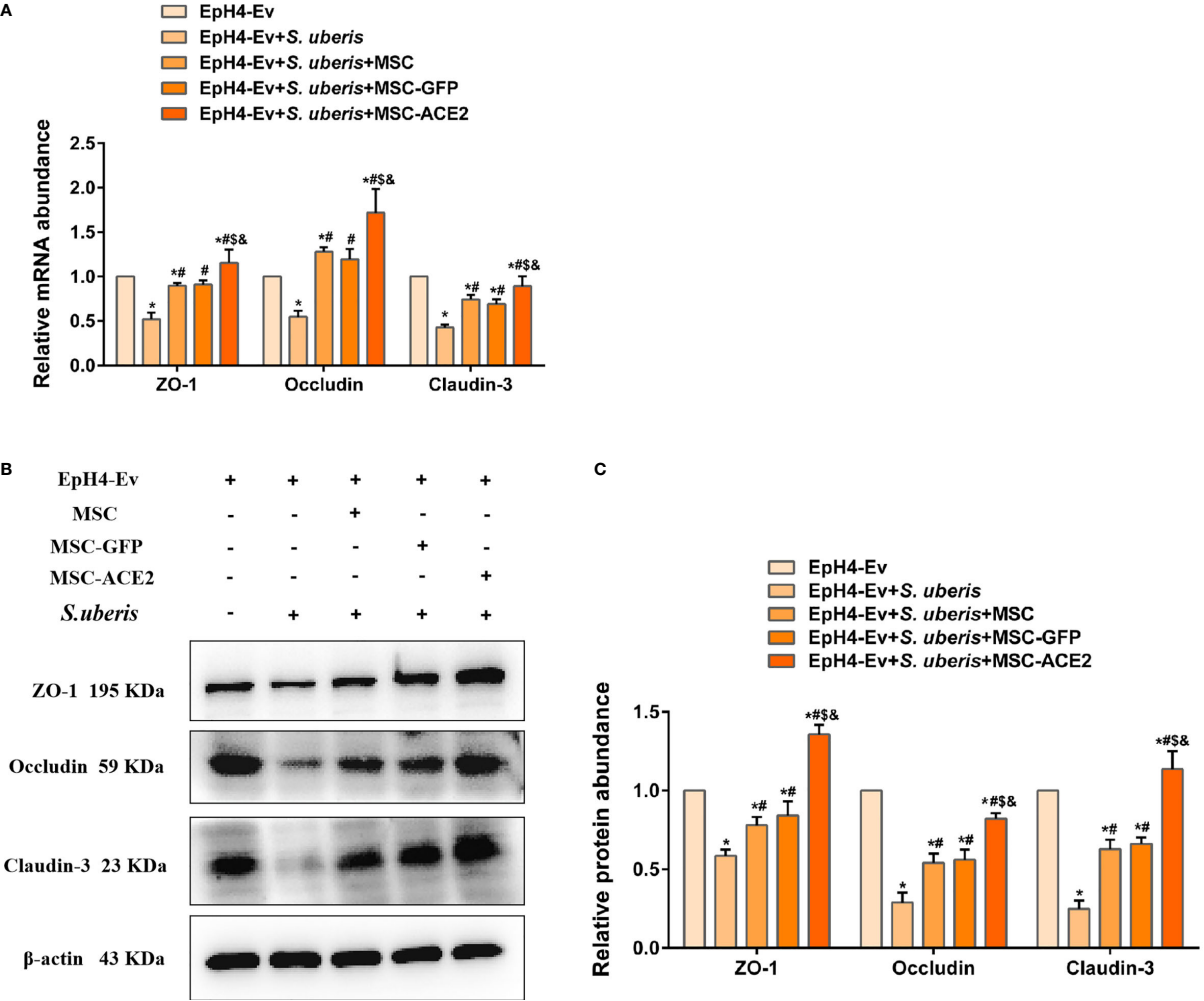
MSC-ACE2 reversed the *S. uberis*-induced downregulation of the expression abundance of blood-milk barrier-associated proteins. **(A)** Detection of relative transcript levels of ZO-1, Occludin, and Claudin-3 in EpH4-Ev cells by qPCR (n = 4). **(B)** Detection of relative protein expression levels of ZO-1, Occludin, Claudin-3 by Western blot; **(C)** Statistics of the ZO-1, Occludin, Claudin-3 Western blot results (n = 3). Experiments were repeated three times and data were presented as the mean ± SEM. ^*^ *P* < 0.05 vs. EpH4-Ev; ^#^ *P* < 0.05 vs. *S. uberis*; *^$^P* < 0.05 vs. MSC; ^&^ *P* < 0.05 vs. MSC-GFP.

As shown in **Figure 8A**, *S. uberis* infection significantly downregulated Occludin, ZO-1, and Claudin-3 transcript levels contrasted with the control group. Conversely, MSC-ACE2, MSC-GFP, and MSC reversed the down-regulation of transcript levels of Occludin, ZO-1, and Claudin-3 caused by *S. uberis*. Moreover, the MSC-ACE2 group had a more significant effect compared with the MSC-GFP and MSC groups. The Western blot results were consistent with the qPCR results, *S. uberis* infection significantly downregulated the expression levels of Occludin, ZO-1, and Claudin-3 compared to the control group. However, MSC-ACE2, MSC-GFP, and MSC all significantly reversed the downregulation of Occludin, ZO-1, and Claudin-3 expression levels due to *S. uberis*, with MSC-ACE2 being the most potent (**Figures 8B, C**).

## Discussion

Mastitis is a common disease in dairy cows that can lead to inflammatory damage to the mammary gland and cause substantial economic losses to the dairy industry worldwide (26, 27). *S. uberis* is one of the important causative agents of mastitis in dairy cows and is the main cause of subclinical chronic mastitis (5). The current treatment of mastitis in dairy cows still relies on antibiotics (28). However, long-term use of antibiotics can lead to problems such as antibiotic residues and bacterial resistance (29). Therefore, there is an urgent need to find new molecular targets and approaches for mastitis treatment.

In this study, we found that *S. uberis* infection of EpH4-Ev cells significantly upregulated the secretion levels of IL-6, TNF-α and IL-Iβ, and significantly activated NAGase activity, indicating that *S. uberis* infection led to the inflammatory injury in EpH4-Ev cells. Conversely, *S. uberis* infection of EpH4-Ev cells upregulated Ang II levels, while downregulating Ang-(1–7) levels. This suggests that inflammatory injury in EpH4-Ev cells due to *S. uberis* is associated with an imbalance of ACE2, Ang-(1–7), and Ang II. However, our results exhibited that MSCs overexpressing ACE2 suggestively upregulated Ang-(1–7) levels while suppressing *S. uberis*-induced upregulation of Ang II, IL-6, TNF-α, IL-Iβ, and NAGase activity. Based on these results, MSCs combined with ACE2 can ameliorate inflammatory injury caused by *S. uberis* in mammary epithelial cells. This suggests that MSCs combined with ACE2 are more effective in alleviating inflammatory damage in mammary epithelial cells induced by *S. uberis*.

Recent studies have shown that MSCs have significant antimicrobial potency (30–33). Similarly, our study found that MSC, MSC-GFP, and MSC-ACE2 treatments all significantly reduced the load of *S. uberis* in EpH4-Ev cells, indicating that MSC, MSC-GFP, and MSC-ACE2 have significant antimicrobial efficacy. Interestingly, MSC combined with ACE2 had greater antimicrobial efficacy than MSC alone. It is worth mentioning that some studies have demonstrated that ACE2 can promote the secretion of antimicrobial peptides and regulate intestinal flora (34, 35). This may be the reason for the more significant antimicrobial efficacy of MSC-ACE2 compared to MSC alone.

An increasing number of studies have shown that MSC has anti-apoptosis and cell proliferation-promoting effects (36–39). Apoptosis is divided into extrinsic (death receptor-mediated) and intrinsic (mitochondria-dependent) apoptotic pathways, which trigger the activation of downstream effector Caspase-3 through a series of signal transduction and ultimately initiate apoptosis (40–42). Fas bind to fatty acid synthetase ligand (FasL), which sequentially activates Caspase-8 and Caspase-3, eventually triggers apoptosis (43). The mitochondria-dependent apoptotic pathway is mainly mediated by the Bcl2 protein family and cytochrome C. Activated Bax (one of the important pro-apoptotic proteins in the Bal-2 family) and leads to an increase in cytochrome-C entering the cytoplasm to promote apoptosis (44–46). However, Bcl-2 inhibits Bax activity and thus exerts anti-apoptotic effects (44). The results from this study indicate that MSC-ACE2, MSC-GFP, and MSC effectively alleviate the *S. uberis*-induced apoptosis in EpH4-Ev cells by upregulating the expression of Bcl2 and inhibiting the expression of Bax and Caspase-3. These results suggest that MSC-ACE2 has a stronger anti-apoptotic effect.

Pyroptosis is an inflammation-associated programmed cell death mediated by members of the gasdermin family, which is accompanied by cell membrane perforation and the release of IL-18 and IL-Iβ (47). Recent studies have shown that streptococcal lipid toxins lead to tissue injury by inducing pyroptosis (48). In the present study, *S. uberis* infection significantly upregulated the expression levels of NLRP3, ASC, cleaved Caspase-1 (p20), cleaved GSDMD, and promoted the release of IL-18 and IL-Iβ, which indicated that *S. uberis* infection caused EpH4-Ev cells to pyroptosis. Interestingly, MSC-ACE2 significantly inhibited *S. uberis*-induced pyroptosis compared to MSC-GFP and MSC, indicating that the combined effect of MSC and ACE2 was more effective. This may be one of the ways in which MSC-ACE2 ameliorates *S. uberi*-induced inflammatory damage in EpH4-Ev cells.

Proteins of the NLRP3, NF-κB, and MAPKs signaling pathways are targets for anti-inflammatory drug research (49–51). This study showed that MSC, combined with ACE2, significantly inhibited MAPKs (p-ERK, p-JNK, p-p38), NF-κB (p-p65) and NLRP3 compared with MSC-GFP and MSC groups. IL-10 is an essential anti-inflammatory molecule that plays a vital role in limiting excessive host inflammation (52). IL-10 promotes the transcriptional expression of the target gene SOCS3 by sequentially activating JAK2 and STAT3, which is the key to the anti-inflammatory effect of IL-10 (53–57). Our study found that MSC-ACE2 promoted the expression of IL-10, p-STAT3, and SOCS3 more effectively. Therefore, MSC-ACE2 inhibited the expression of NF-κB, MAPKs, and pyroptosis pathway-related proteins, probably mediated through the IL-10/STAT3/SOCS3 signaling pathway.

The blood-milk barrier is critical to the body’s defense against pathogenic bacteria invasion (58). Mastitis can disrupt the blood-milk barrier, which can further exacerbate pathogenic infections and increase inflammatory injury to the mammary tissue (59). The tight junction-related proteins Occludin, ZO-1, and Claudin-3 are important components of the blood-milk barrier (60). Studies have shown that increasing the expression levels of tight junction proteins can help fight the invasion of pathogenic bacteria, thus, helping to alleviate mammary gland inflammation (61, 62). In this study, we found that *S. uberis* infection significantly downregulated the expression of the tight junction proteins Occludin, ZO-1 and Claudin-3 in mammary epithelial cells, suggesting that *S. uberis* infection disrupts the blood-milk barrier in mammary epithelial cells. Interestingly, MSC-ACE2 more markedly reversed the *S. uberis*-induced down-regulation of Occludin, ZO-1 and Claudin-3 expression levels compared to MSC-GFP and MSC. This suggests that MSC-ACE2 plays an imperative regulatory role in blood-milk barrier repair.

## Conclusions

**Figure 9:**
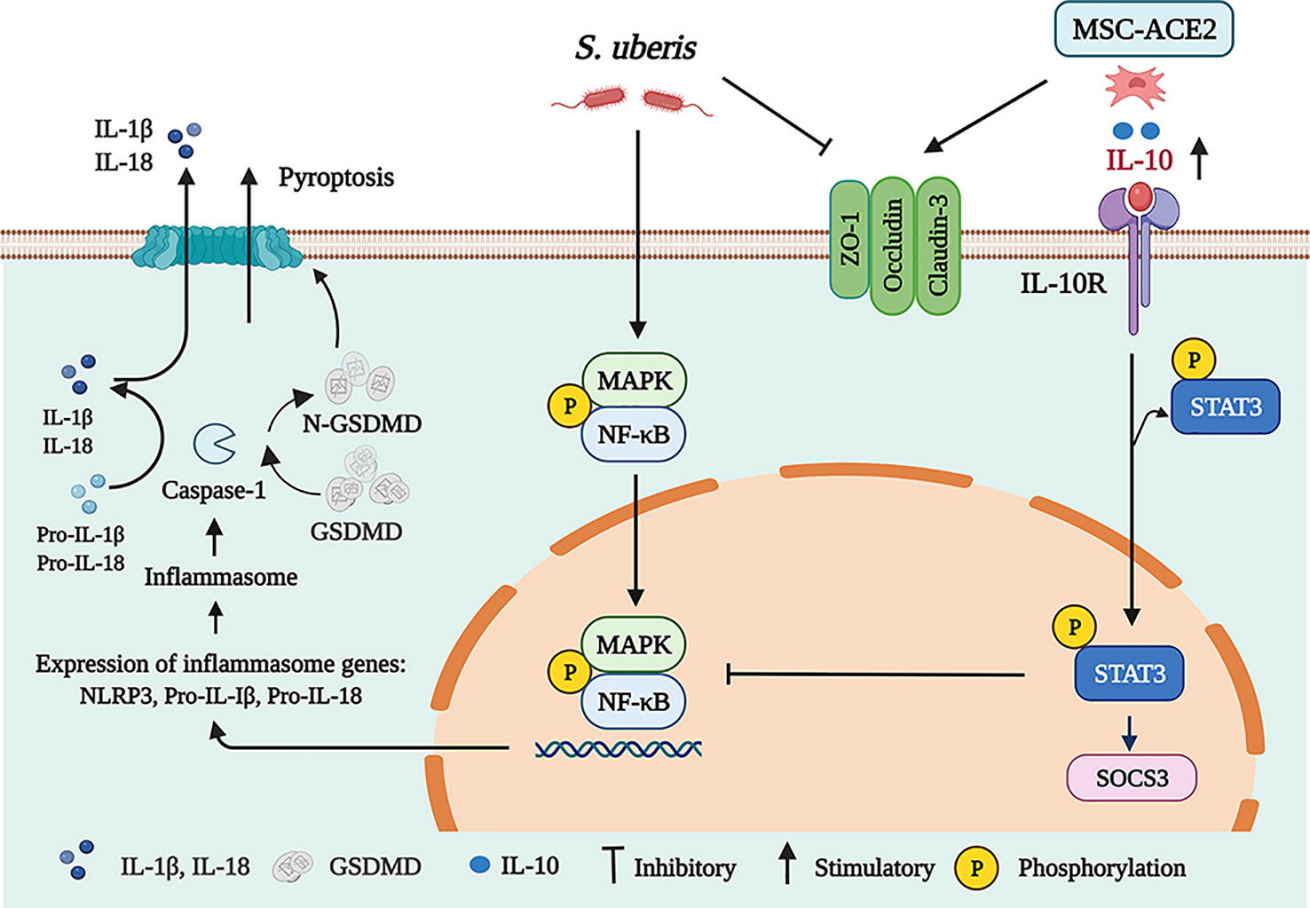
Schematic representation of MSC-ACE2 ameliorate *S. uberis*-induced Inflammatory Injury in Mammary Epithelial Cells by upregulating the IL-10/STAT3/SOCS3 Pathway (Charting with BioRender.com software). Treatment of EpH4-Ev cells with *S. uberis* at MOI = 10 for 3 h caused EpH4-Ev cells pyroptosis, activated MAPKs/NF-κB signaling pathways, promoted the release of inflammatory factors TNF-α, IL-6, IL-Iβ, and IL-18, induced apoptosis, and disrupted the blood-milk barrier. Co-culture with MSC, MSC-GFP or MSC-ACE2 activated the IL-10/STAT3/SOCS3 signaling pathway and inhibited MAPKs/NF-κB, apoptosis, and pyroptosis pathways. Furthermore, MSC-ACE2 reversed the *S. uberis*-induced downregulation of Occludin, ZO-1, and Claudin-3 expression levels and promoted blood-milk barrier repair. MSC-ACE2 had a better effect than MSC alone.

In conclusion, we show that MSC-ACE2 can ameliorate *S. uberis*-induced inflammatory injury in EpH4-Ev cells by upregulating the IL-10/STAT3/SOCS3 signaling pathway and downregulating NF-κB, MAPKs, pyroptosis, and apoptosis signaling pathways. Furthermore, MSC-ACE2 was more effective than MSC alone in reversing the *S. uberis*-induced down-regulation of tight junction protein expression levels in EpH4-Ev cells (**Figure 9**).

## Data Availability Statement

The original contributions presented in the study are included in the article/**Supplementary Material**. Further inquiries can be directed to the corresponding author.

## Author Contributions

Conceptualization, SY and YuZ; methodology, SY and CZ; software, XJ; validation, SY CZ and YaZ; formal analysis, XH; investigation, GW; resources, YuZ; data curation, XJ; writing-original draft preparation, SY; writing-review and editing, SY; visualization, YaZ; supervision, YuZ; project administration, YuZ; funding acquisition, YuZ. All authors have read and agreed to the published version of the manuscript.

## Funding

This research was funded by the National Natural Science Foundation of China, grant number 31972640, and Priority Academic Program Development of Jiangsu Higher Education Institutions.

## Conflict of Interest

The authors declare that the research was conducted in the absence of any commercial or financial relationships that could be construed as a potential conflict of interest.

## Publisher’s Note

All claims expressed in this article are solely those of the authors and do not necessarily represent those of their affiliated organizations, or those of the publisher, the editors and the reviewers. Any product that may be evaluated in this article, or claim that may be made by its manufacturer, is not guaranteed or endorsed by the publisher.
