# Supplementary material for: MSC-ACE2 Ameliorates Streptococcus uberis-Induced Inflammatory Injury in Mammary Epithelial Cells by Upregulating the IL-10/STAT3/SOCS3 Pathway
Source: Front Immunol. 2022 May 23;13:870780. doi: 10.3389/fimmu.2022.870780 (PMC9167935; doi:10.3389/fimmu.2022.870780)
Supplement: Supplementary file 1 [file DataSheet_1.doc]

**Figure 1**

**
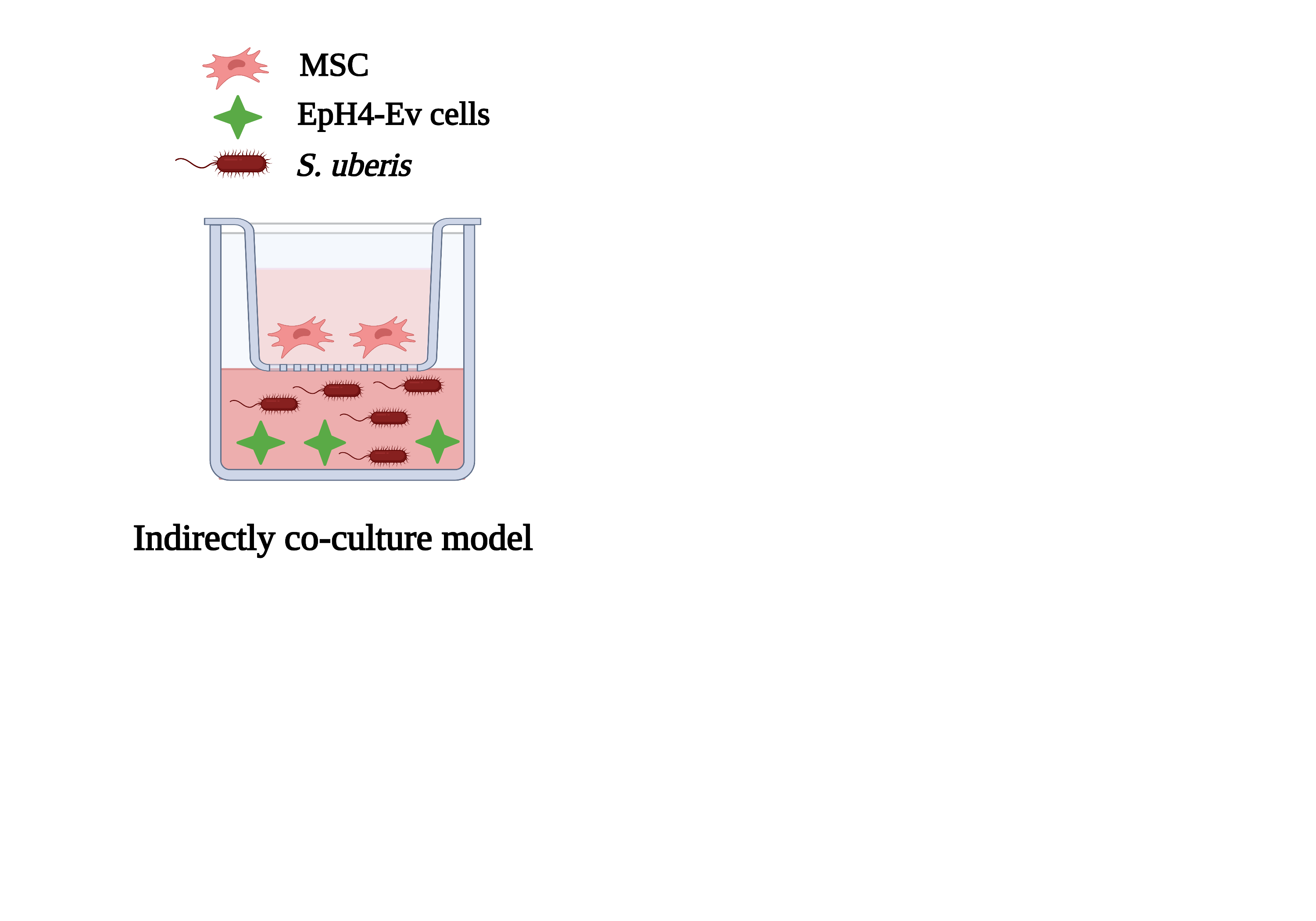
**

**Figure 1. An indirect co-culture model system was used to investigate the therapeutic effects of different MSCs (MSC, MSC-GFP and MSC-ACE2) in *S. uberis*-induced EpH4-Ev cell injury (Charting with BioRender.com software).**

**Table 1 The primer sequences of the genes**

| Target genes | Primer sequences (5’-3’) |
| --- | --- |
| TNF-α | F: TCCCAGGTTCTCTTCAAGGGA |
|  | R: GGTGAGGAGCACGTAGTCGG |
| IL-6 | F: CAAGAAAGACAAAGCCAGAGTC |
|  | R: GAAATTGGGGTAGGAAGGAC |
| IL-10 | F: CCAGGGAGATCCTTTGATGA |
|  | R: CATTCCCAGAGGAATTGCAT |
| IL-Iβ | F: GCCTCGTGCTGTCGGACCCATA |
|  | R: TGCAGGGTGGGTGTGCCGTCTT |
| ASC | F: GAAGTGGACGGAGTGCTGGATG |
|  | R: CTTGTCTTGGCTGGTGGTCTCTG |
| IL-18 | F: GGCCGACTTCACTGTACAACCG |
|  | R: GGTCACAAGCCAGTCCTCTTACTTC |
| SOCS3 | F: GCTCCAAAAGCGAGTACCAGC |
|  | R: AGTAGAATCCGCTCTCCTGCAG |
| ZO-1 | F: GGGAGGGTCAAATGAAGACA |
|  | R: GGCATTCCTGCTGGTTACAT |
| Occludin | F: GTGAGCTGTGATGTGTGTTGAGCT |
|  | R: GTGGGGAACGTGGCCGATATAATG |
| Claudin-3 | F: TTTCTTTGTCCATTCGGCTTG |
|  | R: ACCGTACCGTCACCACTACCA |
| β-actin | F: TCTGGCACCACACCTTCTA |
|  | R: AGGCATACAGGGACAGCAC |
